# Development of a quality of life questionnaire for transgender individuals during hormone therapy (iTransQol)

**DOI:** 10.1007/s00404-022-06689-9

**Published:** 2022-07-25

**Authors:** Katharina Feil, David Riedl, Lisa Gschwentner, Kilian Vomstein, Julia Wegscheider, Emanuela Arnold, Bettina Toth

**Affiliations:** 1 Department of Gynecological Endocrinology and Reproductive Medicine, Medical University Innsbruck, Innsbruck, Austria; 2 University Clinic of Medical Psychology, Medical University of Innsbruck, 6020 Innsbruck, Austria

**Keywords:** Transgender, Quality of life, Questionnaire, Gender affirming hormone therapy

## Abstract

**Purpose:**

Quality of life (QoL) is a complex term, including mental, physical and social health, and everyone’s individual environment. While transgender individuals still often report lower QoL than other individuals, they can benefit substantially from gender affirming therapy.

The aim of this study was to develop a questionnaire to determine QoL in transgender individuals during gender affirming hormone therapy (GAHT).

**Methods:**

A multi-step questionnaire development process was performed. In phase 1, a list of key issues was established by reviewing relevant literature. In phase 2, *n* = 38 transgender individuals as well as *n* = 6 practitioners evaluated the questionnaire (iTransQoL) in terms of relevance, meaning, comprehensibility and redundancy. Psychometric testing of the questionnaire was performed in phase 3 with *n* = 40 transgender individuals. The external validity of the iTransQoL was tested by comparison with three validated health questionnaires.

**Results:**

The exploratory factor analysis indicated an underlying four-factor solution. Psychometric testing showed acceptable to good overall reliability (*α* = 0.73–0.83) for the total score and the four subscales as well as good validity indices. Based on the results, a final version of the iTransQoL was established.

**Conclusion:**

The iTransQoL is a reliable and valid tool to evaluate QoL of transgender individuals during GAHT.

## What does this study add to the clinical work

**Table:** 

Since evaluation of QoL is central to any therapy and a comprehensive questionnaire for recording QoL under GAHT has not been identified, the development of a transgender-specific questionnaire for QoL during GAHT is of utter importance. The iTransQoL provides a valid and reliable questionnaire and allows comprehensive and easy evaluation of the treatment process and adjustment of GAHT if necessary.	

## Introduction

A [1] quality of life (QoL) is seen “as an individual's perception of their position in life in the context of the culture and value systems in which they live and in relation to their goals, expectations, standards and concerns” [2]. The evaluation of QoL comprises at least 4 components: physical, psychological, social and environment [3].

Several studies have described increased levels of depression, suicidal thoughts and a higher rate of discrimination in transgender individuals compared to cisgender individuals [4–8]. QoL in transgender individuals was shown to be decreased before, but improved following gender affirming hormone therapy (GAHT) [6, 9–11].

Gender affirming therapy, like GAHT can improve the well-being and consists of testosterone treatment for transgender men and oestrogen administration for transgender women as well as the suppression of endogenous sex hormone production via antiandrogens or GnRH analogues [12]. GAHT normally continues life long, the antiandrogen or GnRH therapy can be stopped after gonadectomy [12]. The standardized therapy of the Transgender Center Innsbruck includes GnRH analogues in all sexes, as well as therapy with sex steroid hormones. Feminizing GAHT is most commonly performed using 17β-estradiol transdermally; alternatively, patients receive estradiol hemihydrate orally, depending on patient preference and preexisting contraindications. Virilizing GAHT is performed with testosterone undecanoate i. m., less frequently with testosterone transdermally or a combination of both preparations. In Austria, the recommendations of the Ministry of Health for the treatment process of gender dysphoria or transsexualism apply, which were last revised in 2017 [13]. The diagnostic process should include psychiatric, psychotherapeutic and psychological assessment. A consensus decision or positive opinion from the case-managing psychiatrist is a basic requirement prior to body-modifying therapy and required for a change of personal status and name, as well as for GAHT. Psychotherapeutic support during transition is recommended, but not mandatory.

Therefore, evaluation of QoL is a major point in every hormonal therapy, even more in GAHT, and should include factors like physical, mental and sexual health as well as discrimination. Existing transgender questionnaires only partially include these main aspects, dealing with the diagnosis of gender dysphoria or individual areas of QoL, like the occurrence of mental illnesses or dissatisfaction with one's own body. In an extensive literature research, a comprehensive questionnaire for recording QoL under GAHT could not be identified. Thus, the development of a transgender-specific questionnaire for QoL during GAHT is warranted.

## Materials and methods

The iTransQoL was developed in accordance with the recommendations of the European Organization for Research and Treatment of Cancer Quality of Life Group (EORTC QLG) on questionnaire development [14]. In their framework, the EORTC QLG recommends four phases for questionnaire development: (I) compiling an exhaustive list of relevant issues that cover the domains of interest, (II) constructing a preliminary questionnaire that covers all relevant issues, (III) pilot testing, (IV) large scale international field-testing and validation. Here, we present results from the first three phases of the development process for the iTransQoL. The protocol for the questionnaire development was approved by the local ethics committees of the Medical University of Innsbruck (1220/2019) and a signed informed consent was obtained from each participant.

### Phase 1

The aim of phase 1 was the development of an exhaustive list of QoL issues relevant to transgender individuals during GAHT. A scoping literature review was conducted between March 2017 and June 2017 with a combination of 34 German and English search terms was used in different electronic databases (Medline, Psyndex, Embase, Cochrane Controlled Trials Register and Web of Science). The most frequently used questionnaires were extracted and their content was analysed by a multi-professional team of experienced health care professionals (HCPs). If no issue saturation was reached, additional content was added. A preliminary issue list was constructed to be used in the following phases.

### Phase 2

The aim of phase 2 was to evaluate the preliminary issue list in cognitive interviews with transgender individuals and HCPs. The transgender individuals were recruited at the Transgender Center Innsbruck at the Medical University of Innsbruck. Inclusion criteria were: (a) scheduled or ongoing GAHT, (b) aged at least 18 years old, (c) speaks German fluently, and (e) has no apparent cognitive impairment. The sample of HCPs consisted of assistant and senior physicians as well as psychotherapists, who had at least 3 years of clinical working experience with transgender individuals.

Both transgender individuals and HCPs were asked to rate the importance of all issues on a four-point Likert scale (ranging from 1 ‘*not important at all*’ to 4 ‘*very important*’) and to mark the 25 most important issues. Issues were retained, if (a) the mean issue relevance was rated > 3 points by transgender individuals and/or HCPs; (b) at least > 50% of transgender individuals or HCPs considered the issue a priority, while issues were excluded if (c) < 20% of transgender individuals and HCPs rated the issue as ‘not important at all’.

Additionally, transgender individuals and HCPs were asked to comment on the included issues in terms of relevance, meaning and comprehensibility as well as redundancy and missing content in an open field at the end of the questionnaire. Based on the results of phase 2, the preliminary iTransQoL was constructed.

### Phase 3

In phase 3, the preliminary iTransQoL questionnaire was pre-tested and validated. Inclusion criteria were identical to phase 2. A sample of transgender individuals completed the questionnaire as well as a set of debriefing questions to assess the items comprehensibility, importance and if any item was irritating and to suggest potential new items.

It also included an analysis of the validity and reliability of the iTransQoL. The *factorial structure* was evaluated by calculation of an exploratory factor analysis (EFA; maximum-likelihood with varimax rotation). Scree plots and eigenvalues (< 1.0) as well as content analyses were used to determine the ideal number of extracted factors. *Reliability* was evaluated by analysing internal consistencies (Cronbachs’ α) for the total score and subscales. As a rule of thumb, Cronbach’s *α* > 0.90 is considered excellent, while *α* > 0.80 is considered good, *α* > 0.70 acceptable and *α* < 0.70 questionable or poor.

Finally, *validity* was established by calculation of Pearson correlation coefficients with three external questionnaires: (I) the *SF-36* is a generic QoL questionnaire that consists of 36 items which can be summarized into eight subscales (vitality, physical functioning, bodily pain, general health perceptions, physical role functioning, emotional role functioning, social role functioning, mental health) as well as two overarching domains (physical and emotional health) [15]; (II) the *PHQ-9* is a nine-item single-scale measure to assess the level of depressive symptoms [16]; (III) the *GAD-7* is a seven item single scale questionnaire to assess the general anxiety levels [17]. We hypothesized that we would find higher loadings of the iTransQoL total score and the SF-36 domain scores than for the PHQ-9 and GAD-7 scores. Additionally, we hypothesized that the iTransQoL would correlate stronger with specific subscales of the SF-36. Based on the results of phase 3, the iTransQoL was critically revised to construct a final version of the questionnaire which will be re-evaluated in a larger sample in phase 4.

Statistical analyses were performed with IBM SPSS (v22.0) and SPSS AMOS (v24.0). *P*-values < 0.05 (two-sided) were considered statistically significant.

## Results

### Phase 1: Generation of issues

The results of the literature review showed a lot of different questionnaires that had been used to assess QoL in transgender individuals. The vast majority of questionnaires were pain- or disease-specific and thus not applicable to this study. Only two transgender-specific questionnaires could be identified in our review: the Essen Transgender Quality of Life Inventory (ETLI) [18] and the Utrecht Gender Dysphoria Scale (UGDS) [19]. The content analysis, however, revealed that those questionnaires mainly addressed the diagnosis of gender dysphoria or individual domains of QoL (e.g., prevalence of mental illness, body dissatisfaction), while none were designed to assess a more holistic and comprehensive concept of QoL.

In summary, a total of 40 issues were extracted from the literature. Four questions are based on the ETLI [18], 16 on the UGDS [19], 11 on the ‘Questionnaire to assess the own body’ (FBeK) [20], one on the HADS [21] and eight questions are based on the ‘Questionnaire for social integration’ (FSI) [22].

In a subsequent discussion of the findings in a group of HCP experts, 24 additional issues were added to the list. The issue list included items on the personal attitudes towards one's own trans identity, body perception, sexuality, psychological and social functioning (including family-, partner-, children- and work-related issues) as well as more detailed issues about gender dysphoria that were added for trans men and trans women separately.

Furthermore, a demographic query including the topics gender, sexual orientation, age and nationality, a chronological overview of ones outing and treatment so far, background referring to relationships and the desire of having children, illness, smoking and drinking patterns, drug consumption, current housing situation, education and gender affirming surgery was placed at the beginning of the questionnaire to analyse sociodemographic data in parallel.

### Phase 2: Evaluation of issues and construction of preliminary questionnaire

#### Study population

In total, *n* = 38 transgender individuals and 6 HCPs participated in this phase of the study, which was conducted between August 2019 and January 2020. The study group consisted of *n* = 19 trans men (50.0%), *n* = 16 trans women (42.1%) and *n* = 3 non-binary transgender individuals (7.9%). The majority (*n* = 29, 76.3%) were in GAHT, while the remaining 9 patients (23.7%) were in preparation for GAHT.

The majority of transgender with GAHT, started hormonal treatment in 2019 (*n* = 9) and 2018 (*n* = 9). Only *n* = 9 transgender individuals started GAHT between 2014 and 2017 and *n* = 1 in 2008.

The mean age of the study participants (excl. practitioners) was 29.84 ± 13.33 years (mean ± standard deviation). More than two thirds of individuals have been or are currently in a relationship (*n* = 24, 63.2%), just *n* = 6 (15.8%) are married or have been married. An equal number of participants (15.8%) has at least one child and *n* = 14 (36.8%) mentioned the desire to have children. Nevertheless, only *n* = 3 study participants had undergone fertility preservation before GAHT.

Most of the study participants (*n* = 84.2%) had Austrian citizenship. Germany, Bosnia-Herzegovina and Switzerland were named as other countries of origin.

In summary, *n* = 3 (18.8%) of the 16 trans women had genital surgery and *n* = 3 (18.8%) had undergone breast augmentation. Whereas *n* = 7 of the 19 trans men (36.8%) had a mastectomy and *n* = 3 (15.8%) hysterectomy and salpingo-oophorectomy (none had external genital surgery). Non-binary transgender individuals did not have any gender affirming surgery, though all 3 did mention the desire for at least one of the listed surgeries.

### Questionnaire

**Fig. 1:**
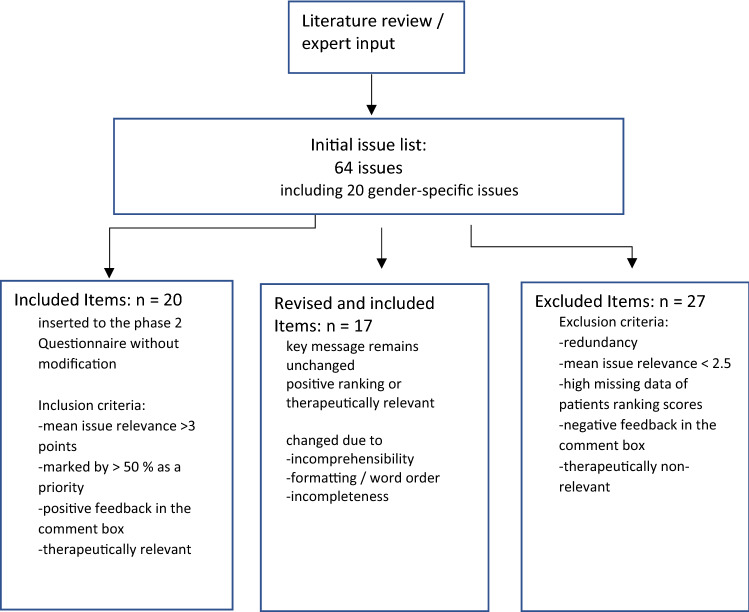
Flowchart on questionnaire development. *HCP* Health Care Professionals

Out of the 64 items that had been identified in phase 1, 20 items were transferred to the developed questionnaire without modification (Fig. 1). 17 had been revised and 27 items were removed due to redundancy or poor rating by the study participants (Tables 1 and 2). 1 new question was introduced based on an HCP’s comment in the debriefing interview. This question refers to the satisfaction with the current voice.

**Table 1:** Importance of iTransQoL issues rated by transgender individuals and HCP

	Issues	Pre GAHT (*n* = 9)		During GAHT (*n* = 29)		HCP (*n* = 6)	
		Mean importance	Priority rating	Mean importance	Priority rating	Mean importance	Priority rating
1	A person’s appearance says a lot about them	3.3	33.3%	3.1	20.7%	2.6	0.0%
2	My body is my home	3.1	22.2%	3.3	44.8%	3.4	33.3%
3	I am sometimes mad at my body	3.2	55.6%	3.3	41.1%	3.7	50.0%
4	My body makes me proud	2.3	11.1%	2.9	27.6%	3.0	0.0%
5	I am satisfied with the way I look	2.4	33.3%	3.1	48.3%	3.4	33.3%
6	I am confident about my trans identity	3.9	55.6%	3.8	62.1%	3.5	50.0%
7	I am able to live my trans identity in public	3.4	77.8%	3.6	69.0%	4.0	66.7%
8	Sometimes my appearance prevents me from being in contact with other people	3.4	22.2%	2.7	44.8%	3.5	50.0%
9	When someone speaks badly about my appearance, I feel hurt	2.5	22.2%	3.1	41.4%	2.3	0.0%
10	I feel happy and carefree	2.6	22.2%	2.7	37.9%	3.2	16.7%
11	I am afraid that I will regret my decision to live out my trans identity	1.8	33.3%	2.0	34.5%	3.8	66.7%
12	I think the healthcare system supports me well	3.4	33.3%	3.7	44.8%	3.0	50.0%
13	I am satisfied with the therapy so far	3.6	44.4%	3.9	62.1%	3.7	66.7%
14	I suffer from side effects of the therapy	1.9	33.3%	2.4	37.9%	3.8	66.7%
15	I find myself attractive	2.9	33.3%	3.2	37.9%	2.7	16.7%
16	I like to be touched	2.3	22.2%	2.9	31.0%	2.5	16.7%
17	I can enjoy my physical sensations during sexual intercourse	2.2	11.1%	2.7	31.0%	2.7	16.7%
18	I can enjoy sexual intercourse overall	2.4	22.2%	2.7	27.6%	2.3	50.0%
19	My bodily situation restricts my sexuality	3.0	44.4%	3.2	41.4%	3.7	66.7%
20	I am satisfied with my sex life	2.1	22.2%	3.1	13.8%	3.5	50.0%
21	My social environment supports me	3.6	66.7%	3.6	62.1%	4.0	83.3%
22	My social environment has changed because of my trans identity	2.6	22.2%	3.0	31.0%	3.0	16.7%
23	I was discriminated against because of my trans identity	2.7	33.3%	2.9	44.8%	3.7	83.3%
24	I can enjoy social events carefree without being restricted by my trans identity	2.8	11.1%	3.3	41.4%	2.7	0.0%
25	I feel uncomfortable in the presence of others	2.3	0.0%	2.5	27.6%	2.7	16.7%
26	I feel lonely	2.3	11.1%	2.5	31.0%	3.3	33.3%
27	I have been suffering from depression lately	2.2	55.6%	2.6	37.9%	3.7	50.0%
28	I have thoughts about suicide	2.0	33.3%	2.3	41.4%	4.0	100.0%
29	I have already attempted suicide	2.0	33.3%	2.7	37.9%	3.6	50.0%
30	I feel supported by my family	3.3	44.4%	3.3	44.8%	3.8	83.3%
31	I avoid contact with my family	2.0	33.3%	2.6	31.0%	3.2	16.7%
32	My work colleagues know about my trans identity	3.3	44.4%	3.2	44.8%	3.3	50.0%
33	I can do the job I want to do despite my trans identity	3.0	33.3%	3.3	34.5%	3.5	50.0%
34	I feel integrated into my work environment	3.1	33.3%	3.3	31.0%	3.2	16.7%
35	I feel respected in my work environment	3.4	22.2%	3.4	37.9%	3.6	33.3%
36	I am interested in dating	3.0	44.4%	2.4	20.7%	2.2	0.0%
37	I currently live in a partnership	2.7	44.4%	2.7	27.6%	3.8	66.7%
38	I do not feel supported by my partner	2.1	22.2%	2.1	6.9%	3.7	66.7%
39	I feel I am not supporting my partner	1.3	0.0%	2.2	10.3%	3.2	16.7%
40	I can talk to my partner about feelings and problems	3.1	44.4%	3.2	20.7%	3.7	33.3%
41	Recently, my relationship status has changed	2.4	0.0%	2.0	13.8%	3.4	33.3%
41.1	If yes, was your trans identity the reason?	2.0	11.1%	2.0	6.9%	3.6	33.3%
42	I feel like I have let my children down	2.6	11.1%	1.8	6.9%	3.2	33.3%
43	My children accept me as before	2.1	22.2%	2.3	24.1%	4.0	66.7%
44	The relationship with my children has changed	2.0	11.1%	2.1	10.3%	3.8	66.7%

	Gender-specific Issues	Trans men pre GAHT (*n* = 8)		Trans women during GAHT (*n* = 11)		HCP (*n* = 6)	
		Mean	Priority (%)	Mean	Priority (%)	Mean	Priority (%)
1	I prefer to act like a man	3.8	50.0%	3.6	18.2%	3.8	16.7%
2	I feel hurt when I am treated like a woman	3.9	62.5%	3.3	18.2%	3.8	16.7%
3	I do not like myself because of my female body	3.6	75.0%	3.6	9.1%	4.0	16.7%
4	I wish I had been born as a man	4.0	50.0%	3.9	36.4%	3.8	33.3%
5	I hate having breasts	3.8	62.5%	4.0	27.3%	3.8	16.7%
6	I hate to experience menstruation, because it makes me feel like a woman	3.8	75.0%	4.0	27.3%	3.8	33.3%
7	My life is only worth living as a man	3.3	50.0%	3.8	18.2%	3.3	16.7%
8	I like to behave sexually like a man	3.5	50.0%	3.6	27.3%	3.4	16.7%
9	I like to be treated like a man at all times	4.0	62.5%	4.0	27.3%	3.3	0.0%
10	I like living a man´s life	4.0	62.5%	4.0	27.3%	3.5	0.0%

		Trans women pre GAHT (*n* = 1)		Trans women during GAHT (*n* = 15)		HCP (*n* = 6)	
		Mean	Priority (%)	Mean	Priority (%)	Mean	Priority (%)
11	I always feel uncomfortable when I act like a man	4.0	100.0%	3.2	26.7%	3.8	16.7%
12	I feel hurt when someone treats me like a man	4.0	100.0%	3.4	33.3%	3.8	0.0%
13	I do not like myself because I have a male body	4.0	0.0%	3.1	13.3%	4.0	33.3%
14	I wish I had been born a woman	4.0	100.0%	4.0	33.3%	3.6	16.7%
15	I do not like to have erections	4.0	0.0%	3.4	26.7%	3.5	16.7%
16	My beard growth bothers me, because it makes me look like a man	4.0	0.0%	3.4	26.7%	3.5	33.3%
17	Only as a woman, is my life worth living	4.0	0.0%	3.4	13.3%	3.4	16.7%
18	I like my sexual role as a woman	3.0	0.0%	3.5	33.3%	3.7	16.7%
19	I would prefer to be treated as a woman by everyone	4.0	0.0%	3.1	33.3%	3.6	0.0%
20	I like living as a woman	4.0	100.0%	3.7	46.7%	3.6	0.0%

Issue list for the iTransQoL with mean importance rating (range 1–4 score points; higher scores indicates higher importance) of patients pre- and during GAHT and HCPs. Additionally, the row ‘priority rating’ includes the percentage of participants who rates the individual item as one of the 25 most important items of the issue list. *HCP* Health Care Professionals, *GAHT* gender affirming hormone therapy, issues in bold remained unchanged; underlined issues were revised

**Table 2:** Revised issues

Initial Issue number—Phase 1	Revised issues
#0 6	I am self-confident about my trans identity
#0 10	I feel happy
#0 27	I have suffered from depression in recent weeks
#0 32	My colleagues at work/school know about my trans identity
#0 35	I feel respected at my work/school environment
#0 38	I feel supported by my family
#0 43	My children accept me as I am
	Revised Issues—Gender-specific
#0 5	My breasts are bothering me
#0 6	My menstruation bothers me
#0 9	I would like to be treated like a man by everyone
#0 11	I prefer to act like a woman
#0 12	I feel hurt when I am treated like a man
#0 13	I do not like myself because of my male body
#0 15	Having erections bothers me
#0 16	My beard growth bothers me
#0 17	My life is only worth living as a woman
	Additional Issues
#0 23	If so, by whom?
	New Issues
	I am satisfied with my voice

The demographic query at the beginning of the questionnaire was revised. Questions referring to fertility preservation and side effects of GAHT were added.

In total, the established questionnaire consists of 38 questions, divided into 22 general questions, 2 additional questions addressed to transgender individuals with a partner or children, 7 trans men-specific questions and 7 trans women-specific questions. The general questions focus on the issues of body image, treatment and its consequences, sexuality, discrimination, mental health and social surroundings. The gender-specific questions query the body image in detail as well as feelings about the birth sex. The trans men and the trans women interview exists of identical questions, adapted to the respective gender.

### Phase 3: Pilot-testing and preliminary psychometric testing

#### Study sample

A total of 40 transgender individuals was recruited for phase 3. The sample consisted of 23 (57.5%) trans men, 16 (40.0%) trans women and one (2.5%) non-binary transgender person. The mean age was 24.18 ± 6.75 years (mean ± standard deviation) and the majority (*n* = 34; 85.0%) underwent GAHT.

#### Factor analysis

An exploratory factor analysis (maximum-likelihood with varimax rotation) was conducted to evaluate the factorial structure of the iTransQoL. After critical evaluation of the initial factor analysis, item 2 (‘*The appearance of a person says a lot about them*’) was excluded from the scale and further analyses as it did not clearly load on a factor and was too generic by nature. While item 11 (‘*I suffer from side effects of the therapy*) neither loaded clearly on a factor, it was dismissed from the factor analysis and thus not attributed to a subscale, but still retained in the questionnaire since adverse events are a crucial influence on patients QoL.

And finally, item 20 (‘*My work/study colleagues know about my trans identity*) was also excluded from the factor analysis due to unclear loading, but retained in the overall score.

Factor analysis was repeated for the remaining 19 items. Bartlett’s test of sphericity (*χ*^*2*^ (231) = 450.8, *p* < 0.001) was significant and the Kaiser–Meyer–Olkin measure verified the sampling adequacy for the analysis (KMO = 0.57). Eigenvalues, scree-plot and content analysis indicated a four-factor solution, explaining 60.6% of the variance. The content analysis for naming the extracted factors was independently conducted by three researchers, differences were resolved by consensus (reconciliation process).

Based on the items content, factor 1 was named ‘*personal and emotional well-being*’, factor 2 ‘*social and occupational support*’, factor 3 ‘*body image*’ and factor 4 ‘*self-confidence*’.

#### Reliability

Good internal consistencies for the total score (*α* = 0.83) and the subscales ‘*social and occupational support’* (*α* = 0.83) and ‘*self-confidence*’ (*α* = 0.80) and acceptable values for the subscales ‘*personal and emotional well-being*’ (*α* = 0.79) and ‘*body image*’ (*α* = 0.73).

#### Validity

As hypothesized, we found higher loadings of the iTransQoL total score and the SF-36 domain scores as well as specific subscales of the SF-36 than for the PHQ-9 and GAD-7 scores.

We found a significant positive correlation between the factor ‘*personal and emotional well-being*’ and the SF-36 subscales ‘*general health perceptions, physical role functioning, emotional role functioning, social role functioning and mental health*’ (Table 3). The same factor showed a negative correlation to GAD-7 and PHQ-9.

**Table 3:** Correlations of the iTransQoL total score and subscales with the SF-36 subscales, PHQ-9 and GAD-7

	SF-36: Vit	SF-36: PF	SF-36: PA	SF-36: GHP	SF-36: PRF	SF-36: ERF	SF-36: SRF	SF-36: MH	PHQ-9	GAD-7
iTransQoL										
Total score	− 0.55***	− 0.23	− 0.37*	− 0.60***	− 0.08	− 0.45**	− 0.54***	− 0.50***	0.49**	0.51**
PEW	− 0.40**	− 0.30	− 0.40**	− 0.60***	− 0.57***	− 0.58***	− 0.69***	− 0.64***	0.56***	0.58***
SOS	0.09	0.07	0.19	− 0.17	− 0.30	− 0.07	− 0.04	− 0.14	0.16	0.03
BI	− 0.26	− 0.30	− 0.26	− 0.35*	− 0.20	− 0.39*	− 0.33*	− 0.33*	0.34*	0.23
SC	− 0.03	0.20	− 0.31*	− 0.41**	− 0.38*	− 0.33*	− 0.13	− 0.23	0.28	0.34*

*PEW* personal and emotional well-being, *SOS* social and occupational support, *BI* body image, *SC* social confidence, SF-36: *Vit* vitality, *PF* physical functioning, *PA* bodily pain, *GHP* general health perceptions, *PRF* physical role functioning, *ERF* emotional role functioning, *SRF* social role functioning, *MH* mental health

*p < .05, **p < .01, ***p < .001

For the factor ‘*personal and emotional well-being*’ a correlation was present for several subject areas: the two categories with the highest Pearson's correlation are ‘emotional role functioning’ and ‘psychological well-being’ from the SF-36 Health Questionnaire. In addition, a positive correlation was also found for three other categories of the SF-36: ‘general health perception’, ‘vitality’, and ‘social role functioning’. The GAD-7 and the PHQ-9 correlated negative with the factor ‘*personal and emotional well-being*’.

*‘Dealing with one’s own body image*’ maps onto ‘social functioning’ of the SF-36 with a low Pearson's correlation. ‘Self-confidence’ from factor 4 of the iTransQol correlated with two items from the SF-36: ‘General health perception’ and ‘vitality’.

Based on the results of phase 3, the iTransQoL was revised, one item was dismissed and a final version of the questionnaire was created.

## Discussion

The objective of this study was to to develop and validate a questionnaire to determine quality of life in transgender individuals before and during GAHT. According to international guidelines the questionnaire was developed by literature review, validation of created items by transgender individuals (trans women, trans men and gender non-conforming) with and before GAHT as well as practitioners, pilot-testing and psychometric testing. During the first phase of the study a preliminary list of 64 issues was created, in phase 2 the issues were reduced to 38 items, during phase 3 one more item was dismissed, so that the final questionnaire consists of 37 items. The scale was developed in German. Our analyses showed good overall reliability. Validity of the iTransQoL was tested by comparison with SF-36, GAD-7 and PHQ-9 and showed not only a good correlation of the total score of iTransQoL, but in particular, high correlations between the factor ‘*personal and emotional well-being’* and the subscales ‘*general health perceptions, physical role functioning, emotional role functioning, social role functioning and mental health*’ of SF-36, thus indicating that the iTransQoL is a reliable instrument to measure QoL of transgender people during GAHT. As it takes approximately 10 min to complete the scale, it comprises a brief and easy method to follow-up progress under GAHT and adapt therapy where necessary.

One method to record gender dysphoria is the “Utrecht dysphoria scale”, which was created to evaluate the effects of gender reassignment and the intensity of gender dysphoria in 12 items [19]. It was recently revised to measure gender identity and comfort with affirmed gender identity for all gender identities and ages [23]. Gender identity can further be evaluated with the “Genderqueer Identity Scale”, which aims to evaluate the social construct of gender binary, theoretical knowledge about gender and gender fluidity [24].

As far as we know, there are only two other questionnaires to measure transgender-specific QoL and not only gender dysphoria or incongruence. The Essen Transgender Quality of Life Inventory (ETLI) is a questionnaire in German consisting of 30 items and aims to measure QoL for the last 4 weeks as well as QoL retrospective at the time of coming out [18]. It is is widely used in German speaking countries and contains questions about trans identity awareness and trans identity specific QoL. The authors state that assessment of transition processes and changes in QoL can help to detect individuals who are in need for counselling. Validation of the ETLI was conducted by testing trans women after gender affirming surgery. The ETLI was not validated for trans men or gender non-conforming individuals under GAHT.

The Gender Congruence and Life Satisfaction Scale (GCLS) is a questionnaire in English consisting of 38 items, aiming to measure improvement of gender (in) congruence, mental well-being and life satisfaction during the last 6 months [25]. This scale was developed to fit for every gender, not being restricted to a binary gender system. The GCLS is currently not validated for languages other than English.

In contrast to the iTransQoL, neither the ETLI, nor the GCLS contain items regarding QoL and satisfaction under GAHT. GAHT is an important milestone for many transgender individuals on their way towards their desired sex. Due to different side effects (e.g., acne, erythrocytosis, thrombosis), it should be monitored and adjusted on an individual basis. Evaluation of QoL in course of gender affirming therapy may give a valuable insight in long-term outcome, factors influencing well-being and possible support during transition.

Fertility preservation is another important issue which is only addressed in the iTransQoL. Since most transgender individuals start gender affirming therapies during reproductive years, the possibility of fertility preservation before GAHT or before gonadectomy at the latest should be offered. Fertility preservation mainly consists of sperm, oocytes or ovarian cryopreservation [12, 26]. Raising awareness for the different options concerning fertility preservation in transgender people and health care professionals is an important goal and can be gained by including fertility preservation questions in commonly used scales.

A limitation to this study is the heterogeneity of the study groups consisting of both, persons before and during GAHT as well as before and after gender affirming surgery. However, the iTransQoL will be further tested in phase 4 in a larger sample, as well as longitudinally before and during GAHT.

In summary, the iTransQoL provides a valid and reliable questionnaire and allows comprehensive and easy evaluation of the treatment process and adjustment of GAHT if necessary.
